# An Algorithm for Identifying Novel Targets of Transcription Factor Families: Application to Hypoxia-inducible Factor 1 Targets

**DOI:** 10.4137/cin.s1054

**Published:** 2009-03-04

**Authors:** Yue Jiang, Bojan Cukic, Donald A. Adjeroh, Heath D. Skinner, Jie Lin, Qingxi J. Shen, Bing-Hua Jiang

**Affiliations:** 1 Lane Department of Computer Science and Electrical Engineering; 2 Mary Babb Randolph Cancer Center, and Department of Microbiology, Immunology and Cell Biology, West Virginia University, Morgantown, WV 26506, U.S.A; 3 Department of Biological Sciences, University of Nevada, Las Vegas, NV 89154, U.S.A

## Abstract

Efficient and effective analysis of the growing genomic databases requires the development of adequate computational tools. We introduce a fast method based on the suffix tree data structure for predicting novel targets of hypoxia-inducible factor 1 (HIF-1) from huge genome databases. The suffix tree data structure has two powerful applications here: one is to extract unknown patterns from multiple strings/sequences in linear time; the other is to search multiple strings/sequences using multiple patterns in linear time. Using 15 known HIF-1 target gene sequences as a training set, we extracted 105 common patterns that all occur in the 15 training genes using suffix trees. Using these 105 common patterns along with known subsequences surrounding HIF-1 binding sites from the literature, the algorithm searches a genome database that contains 2,078,786 DNA sequences. It reported 258 potentially novel HIF-1 targets including 25 known HIF-1 targets. Based on microarray studies from the literature, 17 putative genes were confirmed to be upregulated by HIF-1 or hypoxia inside these 258 genes. We further studied one of the potential targets, COX-2, in the biological lab; and showed that it was a biologically relevant HIF-1 target. These results demonstrate that our methodology is an effective computational approach for identifying novel HIF-1 targets.

## Introduction

In the past decade, we have witnessed unprecedented advances in genomic databases. The completion of the human genome project has provided us with sequence information on human genes, along with their regulatory sequences.^1^ With the large amount of genomic information, developing efficient and effective computational tools to analyze such huge genomic data has become an important challenge. One important application of such analysis is in gene finding. Some programs for gene finding are designed to predict an entire gene sequence.^2^–^6^ However, a majority of them are designed to identify some specific gene segments, such as promoters,^7^,^8^ enhancers,^7^ exons and CpG islands.^8^

Given the special role of transcription factors in gene expression, the identification of transcription factor targets is an important task.^9^–^15^ A transcription factor controls and regulates gene expression by binding to a particular promoter or enhancer region of the gene. DNA fragment lengths for a transcription factor binding vary from 5 to 25 base pairs. However, a larger region of regulatory elements is involved in gene expression. Thus, in addition to the transcription factor binding site, other sequences may play important roles in gene expression. Therefore, more sophisticated approaches need to be explored in order to accurately identify the relevant sequences that control gene expression. Methods based on frequency of *k*-tuples and exhaustive pattern search have been proposed.^14^ Methods that use both global and local alignments to predict transcription factors, and that considers the binding of transcription factors and *cis*-regulatory elements were previously described.^8^,^13^

Suffix tree based methods have been used in pattern discovery problems in biology. While exact pattern occurrences were considered in,^16^ detecting transcription factor binding sites using suffix trees were considered in,^17^,^18^ based on a method for suffix-tree based inexact pattern matching initially described in.^19^ Essentially, inexact (*k*-mismatch) pattern matching was performed progressively: starting from the root, the method performs an exhaustive comparison of all the symbols on each branch that start from the node against the current position in the pattern, until up to *k* positions mismatch on the path, or the pattern is exhausted. The time requirement of the algorithms is exponential with respect to the length of the pattern and the size of the symbol alphabet, which makes the approach impractical for moderately sized sequences, or large number of sequences. In this work, we also use suffix trees as the basis for pattern matching, and consider only exact pattern matching. A key difference in our approach is the consideration of the practical implementation of this important data structure for environments with huge genomic databases, potentially involving millions of sequences, or billions of base pairs.

In this study, we develop a new methodology for identifying novel targets of hypoxia inducible factor 1 (HIF-1) based on the suffix tree data structure. The methodology includes the following four steps. Step1: Construct the suffix tree using a set of promoter sequences from known HIF-1 targets as training genes. Then we extract common patterns that occur in every training gene at least once from the suffix tree. Step 2: Using the common patterns and known HIF-1 binding site sequences to identify all potential HIF-1 target genes from the genome database. Step 3: Process the potential HIF-1 targets by positional analysis to select those targets with predicted HIF-1 DNA binding site and common patterns from above at the 5′ region upstream of the promoter. Step 4: Analyze the accuracy of the prediction for HIF-1 targets. Step 2 and Step 3 together ensure that interested motifs are located only in the 5′ upstream promoter region. This approach may be extended to identify potential novel targets of other transcription factors since they share similar characteristics for binding to the DNA sequence.

We use the suffix tree data structure in the first and second steps.^20^ Given a string *S*[1*..n*] of length *n*, a suffix tree is a rooted tree with *n* leaves, whereby the *i*-th leaf node corresponds to the suffix *S*[*i..n*], each edge in the tree is a substring, and no two edges out of a node start with the same character. There are two advantages in using a suffix tree in complex string matching problems. One is the possibility of finding common patterns from multiple strings in linear time, and the other is the potential to search for multiple patterns in multiple strings in linear time (with respect to the length of the concatenated strings). The storage requirement is also linear. Table 1 lists the popular linear time search algorithms commonly used to search multiple patterns against a sequence (multiple sequences). Each algorithm in the table is described in detail in.^20^ Assume *k* is the number of patterns; *m_i_*(0 < *i* < *k*) is the length of a pattern; *M* is the total length of patterns; *M*’ is the total length of output patterns; *n* is the length of a sequence; *σ*; is the total number of individual character in the sequence.

The Table 1 compares several available string match algorithms when searching with multiple patterns (i.e. set of patterns) against a sequence. From the table, we can see that the suffix tree is the worst with respect to preprocessing time, but it outperforms all the others at the search phase. The *Θ*(*n*) preprocessing and *Θ* (*M*) search of suffix tree is not achievable by any of the other algorithms. The other methods would preprocess each requested string on input, and then take *O*(*n*) or more worst case time to search for the string (*n* can be huge compared to *M* in our case). Thus, in theory, the suffix tree is efficient in both time and space, and has been used in different applications, such as in multiple genome alignment^21^ and in the identification of sequence repeats.^22^ However, there is still the difficulty of practical implementation of suffix trees suitable for analysis of huge datasets. A major contribution of this work is the development of a simple and innovative methodology for using suffix trees, which makes it feasible to use them on large genomic databases. We apply the method to the problem of finding novel targets of HIF-1 transcription factor, using a database containing millions of sequences, or billions of base pairs.

## Materials and Methods

### General methodology

**Figure 1:**
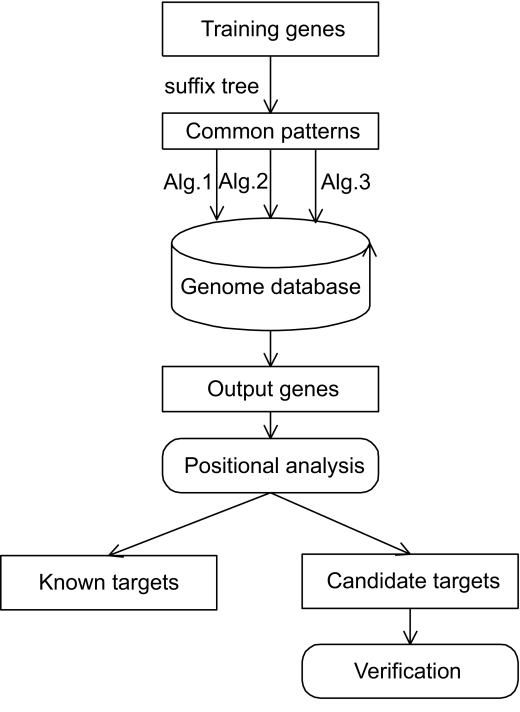
The outline of general methodology. The training genes of known HIF-1 targets are built into a suffix tree, and a set of common patterns are extracted from the suffix tree. Common patterns (including the set of common patterns and consensus sequences) are used to search the human genome database using the suffix tree algorithm. Using positional analysis, we analyze the output genes according to the relative locations of HIF-1 binding sites in the genes, and define the output genes with HIF-1 binding sites upstream of translational start site as potential HIF-1 targets. The potential HIF-1 targets are divided into two groups, known HIF-1 target genes and the candidate target genes. Finally, the candidate novel target genes are validated using available microarray data in the literature and tested in the biological lab.

The general methodology used in this study is illustrated in Figure 1. In brief, 1) A suffix tree is constructed using the set of training genes. A set of common patterns that occur on all training genes at least once is extracted from the suffix tree. 2) Using the multiple patterns (including the common patterns from the previous step and other known patterns such as HIF-1 binding sites (see Table 2) and consensus sequences from the literature, the genome database is searched by applying suffix tree algorithms. This generates the output sequences. 3) Positional analysis is performed on each output sequence according to the functional DNA fragments at the specific locations of the sequence. 4) The output targets from the positional analysis are grouped into known target genes and candidate targets. 5) The candidate target genes are further verified by doing biological experiments in the laboratory and by using available microarray data in the literature.

### Selection of training genes

We used 21 known HIF-1 target genes, and download all available DNA sequences near HIF-1 binding sites from NCBI Nucleotide database (Table 2). In NCBI Nucleotide GenBank, there are gene features for each gene in the annotation database.^45^ We extract 25 different DNA subsequences containing promoter and flanking sequence from these 21 HIF-1 target genes according to the feature information provided in GenBank. The length of subsequence for each HIF-1 target gene training sequence could be different. In these 25 subsequences, there are four genes: HO1, LDHA, EPO, and ENO1 with two different subsequences. Only one subsequence is used for each gene in the remaining 17 HIF-1 target genes. Thus, the known HIF-1 target genes are 21, and the subsequences are 25. We used leave-*k*-out cross-validation method^46^ to select appropriate number of training gene subsequences for this study. Twenty-five HIF-1 gene subsequences are used in this analysis. We denote the 25 HIF-1 target gene subsequences as SET25. The following steps are used: Step 1: 15 training subsequences are randomly selected from SET25. Step 2: these 15 training subsequences are built into a suffix tree and then a set of common patterns that occur at least once in each gene are extracted from the suffix tree. Step 3: these common patterns and HIF-1 binding sites are used to search against SET25. Step 4: the number of the output genes is determined and the accuracy of the approach is calculated. Step 5: Steps 1 to 4 are repeated 1000 times, and the average results are recorded. Similarly, the above procedure is repeated using different numbers of training genes, namely 10, 12, 18, and 20 HIF-1 target gene subsequences. We obtained similar detection accuracy by using 15 and 18 training sequences, and lower detection accuracy using 10 and 12 training sequences. The detection accuracy using 20 training genes is slightly higher. However, the number of common patterns using 20 training genes is much smaller, which could lead to more potential false HIF-1 target genes in the prediction. Thus, we randomly selected 15 training genes in this study. The selected 15 known HIF-1 target gene subsequences are listed and their length of training subsequence are indicated inside parentheses: α_1B_AR(3494), ADM(2356), ALDA(3586), ET-1(1329), ENO1(2312), GLUT1(480), HO-1(908), IGFBP1(1930), LDHA(6166), iNOS(1588), PFKL(699), TFR(365), VEGF(2362), FLT-1(2371), and c-met(3020).

### Suffix tree algorithms for searching genome database

**Figure 2:**
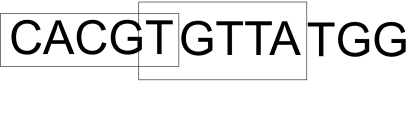
Sliding window method.

To facilitate the practical application of suffix trees on the huge genome database, we use a sliding window method which significantly improved the speed of the algorithms and reduced computer memory requirement. The basic idea is to sequentially analyze smaller chunks of the database based on a chosen window size. Considering a simple example using the string “CACGTGTTATGG” as shown in Figure 2, we wish to determine whether “TT” is in the string. The length of the longest pattern is two in the string. If the machine is able to process five characters at a time, a fixed window of five characters is adopted, and an overlap of one character is needed (overlapping size = the length of longest pattern −1). The window slides from the left to right with the movement size of four characters (movement size = window size—overlapping size). In the first phase, a substring of five characters “CACGT” is read, and used to construct a suffix tree to be searched using the pattern “TT”. In the next phase, the last character “T” from the previous phase is kept, and a substring “TGTTA” should be used to construct a suffix tree. The same process is performed until the search condition is met or the whole string is read.

For a short string, the advantage of using the sliding window may not be obvious. However, the sliding window method becomes extremely important when the string is long and the available computer memory is limited. For example, for large DNA sequences with 5,000,000 base pairs or a concatenation of several DNA sequences, the sliding window method has a noticeable advantage. The sliding window is particularly useful when the whole database (10, 268, 238, 630 base pairs in our case) is needed to be built into a suffix tree. The whole database can be viewed as a large string formed by concatenating all the DNA sequences in the database.

In this section, we describe the algorithms used to search the huge genome database to identify the potential novel target candidates. We use both the common patterns from the training genes (Table 3), and known HIF-1 binding sites (Table 2) as criteria in this search. If a gene contains all the common patterns and one of the HIF-1 transcription factor binding sites, then the gene is selected as an output gene. The stage of searching the huge genome database is a major bottleneck in finding potential novel transcription factor targets. Thus, three algorithms are proposed for this task. We refer to these three algorithms as *Algorithm* 1, *Algorithm* 2 and *Algorithm* 3, respectively.

Algorithm 1 constructs one suffix tree for each sequence, then uses the common patterns to search against each suffix tree. Algorithm 1 is described as follows:

### Algorithm 1

1. set number of characters to be processed *w_s_* = 8000 (note: we assume 8000 characters are processed at one time)
2. compute length of longest common pattern (overlap size).
3. **for** each sequence, *S_i_*, in database **do**
4. set overlap string *O_s_* to empty
5. **while** not end of sequence *S_i_* **do**
6. set *S_tmp_* = |*O*_s_| + *w_s_* characters of *S_i_*
7. construct a suffix tree, *ST*, for the subsequence *S_tmp_*
8. use multiple patterns search against the suffix tree *ST*
9. record the search result
10. determine the content of overlap string *O_s_*
11. update position for next *w_s_* characters from *S_i_*
12. end while
13. end for

Algorithm 2 uses the common patterns to build a suffix tree (*ST_c_*), then uses the individual sequences (*S_i_*) in the database to search against the suffix tree, *ST_c_*.

### Algorithm 2

1. set number of characters to be processed *w_s_* = 8000 (note: we assume 8000 characters are processed at one time)
2. calculate *L_1_*, the length of the shortest pattern among the multiple patterns
3. calculate *L_2_*, the length of the longest pattern among the multiple patterns
4. concatenate all the multiple patterns into one sequence, *S_c_*
5. construct a suffix tree, *ST_c_*, for *S_c_*
6. **for** each sequence, *S_i_*, in database **do**
7. set overlap string |*O_s_*| to empty
8. **while** not end of sequence *S_i_* **do**
9. set *S_tmp_* = *O_s_* + *w_s_* characters of *S_i_*
10. **for** each pattern *P_p_* in *S_tmp_* whose length is from *L_1_* to *L_2_* **do**
11. search *P_p_* against *ST_c_*
12. record the search result
13. end for
14. determine content of overlap string *O_s_*
15. update position for next *w_s_* characters from *S_i_*
16. end while
17. end for

Algorithm 3 builds a suffix tree for the concatenation of all sequences (denoted *ST_d_*), and another suffix tree for a concatenation of the common patterns (denoted *ST_c_*). Then, the suffix tree *ST_c_* is used to search against the suffix tree, *ST_d_*.

### Algorithm 3

1. concatenate all the multiple patterns into one sequence, *S_c_*
2. construct a suffix tree, *ST_c_*, from *S_c_*
3. concatenate all the database sequences into one sequence, *S_d_*
4. construct a suffix tree, *ST_d_*, from *S_d_*
5. use *ST_c_* to search against *ST_d_*
6. record the search result

Algorithm 3 constructs a suffix tree for the entire database and stores it for later search. If Algorithm 3 is applied to a huge database such as the genome database, the suffix tree *ST_d_* is built from all the sequences in the database. Thus, it requires a powerful machine with a huge memory. If we have such a machine that can be used to build a suffix tree for all the database sequences, this algorithm certainly would have some advantages: the whole database only needs to be built into a suffix tree once, and the database can be stored as one big suffix tree. It can be used to search different pattern sets as many times as one may wish. In this case, the search process is very fast, since the time used is linear with respect to the length of concatenated common patterns.

The proposed algorithms utilize the sliding window method to build a suffix tree (except for Algorithm 3). The processed DNA sequence is in FASTA format. A line of FASTA format DNA sequence contains 80 characters except the ending line. Thus, the sliding window algorithm process 100 lines (8000 characters) at a time, for a fixed window size of 8000 characters.

### Positional analysis

Using Algorithm 1 and Algorithm 2, we searched the genome database. The output genes from both algorithms were the same. The only difference was the time each required. We further analyze the output genes using positional analysis.

**Figure 3:**
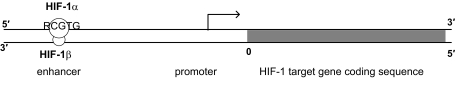
The regulation of a typical HIF-1 target gene. A HIF-1 target gene codes for a specific protein. The promoter is located immediately upstream of the coding sequence for the protein for regulating the gene expression. The enhancer is located upstream of the promoter with different lengths of spacing and with HIF-1 binding site. HIF-1 consists of HIF-1α and HIF-1β subunits. HIF-1α and HIF-1β can dimerize, and bind to the enhancer region to increase its promoter activity. HIF-1 commonly has the binding site “RCGTG” in the enhancer region.^41^,^42^,^44^

A typical schematic diagram of a target gene activated by HIF-1 is shown in Figure 3. It is known that HIF-1 has the consensus binding site “RCGTG” (R stands for any of the four nucleotides: A, C, G, and T) at its target genes.^41^–^44^ All the known HIF-1 binding sites are at the 5′ region upstream of the promoter sequence, that is, in 5′ enhancer region, except erythropoietin (EPO) which contains HIF-1 binding site in the 3′ enhancer region. From the information provided by the annotation databases in GenBank, it is quite difficult to obtain the stop site of gene coding sequence. Therefore, in the positional analysis, we only select the potential HIF-1 candidate targets that contain HIF-1 binding sites in the 5′ region upstream of the promoter.

To identify genes that have the HIF-1 binding site in the 5′ region upstream of the promoter, we need to find the HIF-1 binding site which is in the 5′enhancer region from the target gene sequences. Letting *V_s_* denote 5′ region upstream of the promoter, three methods are used to extract *V_s_* from gene sequence based on the feature tables provided in the GenBank annotation database.^45^ Method 1: For those gene sequences with the available enhancer sequence and position in the feature table, we extract the enhancer DNA sequence as *V_s_*. Method 2: For those gene sequences with the available promoter region and sequence in the feature table, *V_s_* is the DNA sequence of the 5′ region upstream of the promoter plus the promoter region. Method 3: For the remaining gene sequences with no information on either the promoter or enhancer sequence, we search for the first position of the beginning of “CDS”, “TATA” box, or “CAAT” box sequences, called *E_e_*. Then, we extract DNA sequence from 5′ end to *E_e_* as *V_s_*. After determining *V_s_* by using the above three methods, we use Boyer-Moore fast string matching algorithm^20^ to search whether the HIF-1 binding site “RCGTG” is inside *V_s_*.

### Lab verification

Human prostate cancer cells, PC-3 cells were cultured in RPMI 1640 supplemented with 10% fetal bovine serum (Intergen, Purchase, NY), 0.2 units/ml human insulin (Sigma, St. Louis, MO), 50 units/*ml* penicillin, and 50 *mg/ml* streptomycin (Invitrogen, Carlsbad, CA). These cells were seeded in a 12-well plate overnight, and transfected with the indicated plasmids using lipofectamine (Sigma) per the manufacturer’s instructions. Briefly, COX-2 reporter plasmid (0.4 *μg*) containing a 960-bp human COX-2 promoter with the potential HIF-1 binding site was co-transfected with *β*-gal plasmid, and the control vector, HIF-1 dominant negative construct, or HIF-1 *α* expression 1plasmid using 2 *μl* Lipofectamine per well in serum-free Opti-MEM media (Invitrogen, Carlsbad, CA) for 30 *min*. The transfection solution was then added to the cells, and incubated with cells for 4.5 *h*. The cells were then washed and cultured in the medium for 36 *h*. The cells were collected and analyzed using luciferase analysis buffer (Promega, Madison, WI). Luciferase activity was measured using a moonlight luminometer, and *β*-gal activity was measured as a control using the above cellular extracts. The relative luciferase activity was the ratio of luc/*β*-gal with the value normalized to the control as described previously.^27^,^49^

## Results

In this study, we have used HIF-1 target genes as a model system, and developed a new methodology for identifying the novel HIF-1 target genes. Using a training set of 15 known HIF-1 target genes, we have obtained 238 potential HIF-1 targets including 25 known HIF-1 targets from a large genome database. Although suffix trees have been around for some time, the key innovation in our approach is how to use them efficiently on a large database, using a standard personal computer. Our proposed method is particularly efficient, handling a large database of 2,078,786 DNA sequences with a total of 10,268,238,630 base pairs on a PC with 2.8 GHz, and 512 RAM. This confirms the feasibility of the proposed methodology. In addition, through literature search, 17 putative novel targets are verified by microarray data to be upregulated by HIF-1 or hypoxia. We also considered COX-2, one of the potential new targets proposed by our algorithm, and confirmed that COX-2 is a biologically relevant HIF-1 target gene. These results further demonstrate that this new methodology is effective in predicting novel HIF-1 targets.

### Common patterns from training genes

To obtain the common patterns of HIF-1 target genes, we built a suffix tree using the randomly selected 15 known HIF-1 target training genes. From the suffix tree, we extracted a set of 105 common patterns that occurred in all training genes at least once. We fixed the minimum length at 4 base pairs. These are listed in Table 3.

### Comparison of algorithms for searching genome database

The suffix tree data structure is constructed in linear time using Ukkonen’s linear time algorithm.^20^ The three algorithms proposed all have the same overall theoretical running time complexity. Each requires linear time, with respect to the total size of the database (i.e. length of all the concatenated database sequences). We consider the algorithms in terms of the suffix tree construction time, search time using the suffix tree, and memory requirement for the two stages. This is summarized in Table 4.

In terms of running time, the major difference is how much time each algorithm spends in constructing the suffix tree(s), or in searching while using the constructed suffix tree(s). For instance, while Algorithm 1 and 3 spend more time in constructing the suffix tree *O*(*n_s_l_s_*), they spend less time in searching on the suffix tree *O*(*n_p_l_p_*), where *n_s_* = number of sequences in the database, *n_p_* = number of common patterns, *l_s_* = average length of a sequence, and *l_p_* = average length of a pattern. The reverse is the case for Algorithm 2. The overall time complexity (combining tree construction and searching) remains the same for the algorithms.

The memory requirement is, however, quite different for the three algorithms. For Algorithm 2, the advantage is that we only need to build a suffix tree for the multiple patterns once, then use it throughout the whole search. Algorithm 3 for instance requires extra memory proportional to the size of the entire database. It is obvious that Algorithm 2 should be the fastest and most practical if we do not have a powerful machine to support Algorithm 3. This is because, on average, the total length of the common patterns (i.e. after concatenation) is usually shorter than the length of a gene sequence, and the preprocessing time to build the suffix tree is quite short. Moreover, the suffix tree for the common patterns only needs to be built once. In practice, Algorithm 2 is the fastest of the three algorithms, although it has the same space complexity as Algorithm 1.

Algorithm 1 and 2 are more practical for those who do not have a supercomputer with huge memory. For instance, in our case, computational experiments were carried out on a Pentium 4 PC with 2.8 GHz and 512 MB memory. Thus, we implemented Algorithms 1 and 2, and use them to search the genome database.

The nucleotide database was divided into approximately 6 equal parts (based on the number of sequences). Algorithm 1 and Algorithm 2 were executed separately on these 6 parts of the database. The comparative results are shown in the Table 5. As can be observed, in each part of the database, Algorithm 2 processed more DNA sequences and more bytes per minute than Algorithm 1. On average, Algorithm 2 is about 36% faster than Algorithm 1.

### Output genes from genome database

The final output genes after processing for the positional analysis are divided into two groups: the mammalian group contains genes from mammals, such as human, rat and bovine; the other group contains genes from non-mammals, such as virus and plant. Within the potential novel targets, the same gene in different species is counted as one gene. One of the goals is to find genes that may have important implications in human health and disease research. Thus, further analysis of the genes in the mammalian group was conducted. A total of 258 distinct genes were identified.

### Verification of candidate targets

After applying positional analysis to the output genes, the remaining genes are called candidate targets. We further characterize the candidate targets using three approaches: by using known HIF-1 target genes in the literature, by microarray data from literature search, and by biological lab verification.

### Verification of potential novel HIF-1 targets using known HIF-1 targets

In our final output, there are 25 known HIF-1 targets identified. Inside these 25 known output targets, there are 15 HIF-1 targets that are used for the training analysis. Additional six genes in the predicted output were also known HIF-1 targets: cyclin G2, p21(WAF), PGK, TGFα, Nip3, and trefoil factor. These 25 HIF-1 targets are shown in Table 6.

### The validation of candidate novel HIF-1 targets using available microarray data

In a follow-up literature search, additional 17 putative novel HIF-1 targets from the output list were confirmed to be upregulated by HIF-1 or hypoxia by the microarray data. These targets are shown in Table 7. This result showed that our predicted novel HIF-1 targets can be found as upregulated targets of HIF-1 and hypoxia, further confirming the accuracy of our prediction.

### Laboratory validation of a candidate novel HIF-1 target

We selected one of the candidate HIF-1 targets identified as described above to be tested in the biology laboratory. The verified gene was human cyclooxygenase-2 (COX-2) gene. There are two reasons for selecting COX-2. First, COX-2 is important in biological function such as tumor growth and angiogenesis. Second, the availability of COX-2 promoter construct (kindly provided by Dr. Jian Li, Harvard University, MA). It is difficult to obtain promoter constructs for each gene in our final output. COX-2 was a putative target at the time the experiment was carried out (See,^47^ but its regulation by HIF-1 has been recently published independently.^48^

**Figure 4:**
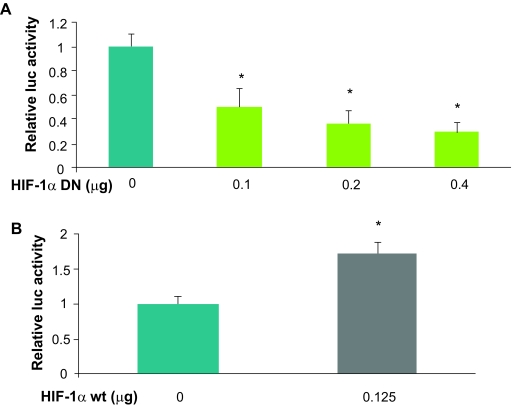
Effect of HIF-1 expression on COX-2 transcriptional activation. PC-3 prostate cancer cells were seeded into 6 well plates a day before the transfection. **a**) To determine whether HIF-1 activity is required for COX-2 transcriptional activation, the cells were co-transfected with COX-2 promoter luciferase reporter (PXP4/COX-2), pCMV-β-gal, and pcDNA3 vector or pcDNA3-HIF-1 dominant negative plasmid. **b**) To determine whether HIF-1 expression is sufficient to induce COX-2 transcriptional activation, the cells were co-transfected with the COX-2 promoter reporter, pCMV-β-gal, and pcDNA3 vector or pcDNA3-HIF-1α wild type expression plasmid. The cells were cultured for 36 h after transfection. The relative luciferase activity was determined by the ratio of luciferase/β-gal activity, and normalized to the vector control (100%). *Indicates the significant difference when the value is compared to the control (*p* < 0.01).

It is known that HIF-1 target genes are regulated at the transcriptional level by triggering their promoter activity. Therefore, to determine whether HIF-1 expression plays a role in COX-2 transcriptional activation, PC-3 prostate cancer cells were transfected with a COX-2 promoter reporter containing a 960-bp human COX-2 promoter with the potential HIF-1 binding site. Expression of HIF-1 dominant negative construct specifically inhibited HIF-1 activity, and inhibited the COX-2 reporter activity in a dose-dependent manner (Fig. 4a). This result indicates that HIF-1 activity is required for COX-2 transcriptional activation. In order to determine whether HIF-1 is sufficient to induce COX-2 transcriptional activation, HIF-1α expression plasmid was co-transfected with the COX-2 reporter. The expression of HIF-1α in PC-3 cells induced HIF-1 expression and COX-2 reporter activity in a dose-dependent manner (Fig. 4b). Thus, HIF-1α is also sufficient to induce COX-2 transcriptional activation. This data demonstrates that COX-2 is a functional HIF-1 target. These result further shows that our methodology is effective in identifying HIF-1 novel targets. Lab verification indicates that HIF-1 is essential in regulating COX-2 transcriptional activation.

While there are certainly many potential HIF-1 targets in the final output, we performed experiments on COX-2. The complete list of output genes is in the supplementary files. We hope that the results of this work will spur others to run the required biological experiments to validate the genes from the final list and to test these potential HIF-1 targets.

## Discussion

The basic methodology in this study is as follows: 1) extract common patterns from the known gene sequences; 2) use the set of common patterns to search the genome database; 3) analyze the target genes according to the specific gene’s feature in the database.

The methodology proposed here is to identify HIF-1 novel target genes using a combination of the specific HIF-1 binding sequence “RCGTG” and the common patterns. Our approach can be applied to other transcription factors. The transcription factors generally have common DNA binding sequences such as activator protein 1 (AP-1),^38^ and nuclear factor-kappaB (NF-*k*B).^39^ AP-1 has the common binding site “TGACTCA”.^54^ NF-*k*B has the common binding site “CAAGGAGGGAA TTCCCGAGT.”^55^,^56^

The methodology may be extended to study other functional genes because many genes are conserved across widely divergent species with similar functions. Genes with similar functions may have similar structure and sequences. Genes belonging to the same family commonly share specific sequences and/or consensus sequences. The idea is to generate the common patterns from known genes, then to use these common patterns to search for unknown novel targets. Thus, steps 1 and 2 may be applied to novel function prediction based on gene structure. We use the annotation database in GenBank which is available to the public. Apart from transcription factors studied here, the databases can be used to study other functional DNA segments, such as exons, introns, miRNAs, and 5′UTRs. For a different kind of gene, step three needs to be changed to adapt to the specific gene’s feature, but the basic idea remains the same.

Furthermore, the approach may potentially be applied to other genes that have known consensus sequences and common regulatory patterns. The suffix tree method can be applied to general gene clustering and classification that needs to group and categorize similar genes together. An improvement in the results (for instance, further filtering the output target genes) could be obtained by combining the proposed suffix tree approach with statistical models.

Although the suffix tree data structure is used for exact string matching in this study, the suffix tree analysis can be further developed for inexact string matching problems.^20^ The inexact matching such as *k-mismatch* is an inexact pattern matching problem: identify all the occurrences of pattern P in text T which allowing k characters of mismatch of pattern P. *k-mismatch* is very useful to find functional similarities (or gene mutations) among genes in bioinformatics.^17^,^18^,^20^ In DNA sequences, mutation, insertion or deletion of nucleotide(s) happens frequently across different species or different individuals where the functional signals may not show up exactly. MicroRNA (miRNA) are a class of small non-coding RNAs with 21 to 23 base pair in length with hairpin structure, that play important roles in regulating post-transcription mRNA expression in animals and plants. Identification of miRNAs using computational methods is successful.^57^ Most of computational prediction of novel MiRNA is based on phylogenetic conservation and structure similarity in closely related species, such as human,^57^,^58^,^60^ animal,^57^,^60^ insect,^57^,^59^ and plants.^57^ It would be interesting and useful to extend this suffix tree method to identify the potential targets of miRNAs in the future study. Taken together, the approach proposed here may be used as a general methodology to identify novel gene targets of a given transcription factor, and to study other gene function and regulation in the future.

## Figures and Tables

**Table 1:** Comparison of common string match algorithms.

Algorithm	Preprocessing	Search
Rabin-Karp	*Θ*(*M*)	*O*(*nM*)
Aho-Corasick	*Θ*(*M*)	*O*(*n + M*’)
Knuth-Morris-Pratt	*O*(*M*)	*O*(*nM*)
Boyer-Moore	*O*(*M* + *σ*)	*O*(*nM*)
Suffix tree	*Θ*(*n*)	*Θ*(*M*)

**Table 2:** The HIF-1 binding sequences from 21 known HIF-1 target genes.

Gene		Subsequences		Ref.
α_1B_AR	5′-CAGGCGA	CGTG	CTGCCGGG-3′	23,24
ADM	5′-CCCGTGGCAAA	CGTG	TTC-3′	24
	5′-GACAAA	CGTG	TCTAGCGTGAT-3′	24
	5′-ACAAA	CGTG	TCTAGCGTGAT-3′	25
ALDA	5′-CCCCCTCGGA	CGTG	ACTCGGACCAC-3′	25
	5′-GA	CGTG	ACT-3′	25
	5′-CTTCA	CGTG	CGGGGACCAGGGACCGT-3′	26
	5′-GGGATGTGGTCCGAGT	CACG	TCCG-3′	26
ET-1	5′-CGGGTCTTATCTCCGGCTG	CACG	TTGCCTGTGGGTGACTAAT CACACAATAA-3′	26
ENO1	5′-GGCCA	CGTG	CGCCGCCTGCGCCTGCG-3′	26
	5′-AGGGCCGGA	CGTG	GGGCCCC-3′	26
	5′-ACGCTGAGTG	CGTG	CGGGACTCGGAGTACGTGACGGA-3′	26
	5′-CGCA	CGTG	GCCCCGGACACGCAGC-3′	26
EPO	3′-GCCCTA	CGTG	CTGTCTCACACAGCCTGTCTGAC-5′	26,27
	3′-GCCCTA	CGTG	CTGTCTCACACAGCCTGTCTGAC CTACCGG-5′	28
	3′-GGGGCTGCTGCAGA	CGTG	CTGTCTCACACAGCCTGTCTGAC-5′	29
	3′-GCCCTA	CGTG	TCTCACACAGCCTGTCTGAC-5′	29
	5′-TGAGACAG	CACG	TAGGGC-3′	30
	5′-GCCCTA	CGTG	CTGCCTCGCAT-3′	26,27
	5′-GCTGGGCCCTA	CGTG	CTGTCTCACACAGCCTGTCT-3′	26,27
	5′-CCTA	CGTG	CTGTCTCACACAGCCT-3′	26,27
GLUT1	5′-TGGGTCCACAGG	CGTG	C-3′	31
	5′-CAGG	CGTG	CCGTCTGACACGCATC-3′	32
HO-1	5′-GAGCGGA	CGTG	CTGGCGTGGCACGTCCTCTC-3′	33
IGFBP1	3′-CAACTA	CGTG	CTCTGG-5′	34
	5′-GCAGGA	CGTG	CTCTGGGGGGCACACATAGCT-3′	34
	3′-TGCCCA	CGTG	CTGGCA-5′	34
	3′-GACACA	CGTG	CTTTCT-5′	34
	3′-GACACA	CGTG	CTTCCT-5′	34
LDHA	5′-ACA	CGTG	GGTTCCCGCACGTCCGC-3′	27
	5′-GTGGGAGCCCAGCGGA	CGTG	CGGGAA-3′	27
	5′-CACA	CGTG	GGTTCCCGCACGTCCG-3′	26
iNOS	5′-GTGACTA	CGTG	CTGCCTAGGGGCCACTGCC-3′	35
	5′-AGTGACTA	CGTG	CTGCCTAGG-3′	28
p35srj	5′-GTGTGCG	CGTG	GTGCCATACGGGACGT- GCAGCTACGTGCCCA-3′	30
FKL	5′-CCGGGTAGCTGGCGTA	CGTG	CTGCAG-3′	24
PGK1	5′-GA	CGTG	ACAAACGAAGCCGCACGTC-3′	27
	5′-CGCGT	CGTG	CAGGACGTGACAAATGGAAGTAG CACGTC-3′	
	5′-GTGAGA	CGTG	CGGCTTCCGTTTG-3′	24
	5′-CTGCCGA	CGTG	CGCTCCGGAG-3′	24
TF	5′-TTCCTG	CACG	TACACACAAGCGCACGTATTTC-3′	36
	5′-GTGTGATTGT	CGTG	GTAGTGGATTCCATGC-3′	36
	5′-A	CGTG	CGCTTTGTGTGTACGTGC-3′	36
TR	5′-AGCGTA	CGTG	CCTC-3′	36
	5′-CGCGAGCGTA	CGTG	CCTCAGG-3′	36
	5′-AGCGTA	CGTG	CCTCAGGAAGTGACG CACAGCCCCCCTG-3′	36
	5′-GGTGTA	CGTG	CGGAAGGAAGTGACGTAGATCCA GAGGG-3′	36
VEGF	5′-CCACAGTGCATA	CGTG	GGCTCCAACAGGTCCTCTT-3′	27
FLT-1	5′-TTGAGGAACAA	CGTG	GAATTAGTGTCATCGTAAAT-3′	37
	5′-TTGAGGAACAA	CGTG	GAATTAGTGTCATAGCAAAT-3′	37
Met	5′-TTAGCGGAGA	CGTG	GGAGAGGCCGAGAG CAAAGCTCGCG-3′	38
	5′-ACCTTGT	CGTG	GGCGGGGCAGAGGCGGGAG GAAACGC-3′-	38
	5′-CAGACA	CGTG	CTGGGGCGGGCAGG-3′	38
	5′-CAGCGCG	CGTG	TGGGAAGGGGCGGAGGGAGTGC-3′	38
	5′-GGAGCGCG	CGTG	TGGTCC-3′	38
Nip3	5′-CCCGCGCACGCGCCGCA	CGTG	CCGCACGCGCCCCGCG-3′	39
RTP801	5′-ACGTTGCTTA	CGTG	CGCCCGG-3′	40

**Abbreviations:** α_1B_AR, α_1B_ adrenergic receptor; ADM, adrenomedullin; ALDA, aldolase A; ET-1, endothelin-1; ENO1, enolase 1; EPO, erythropoietin; GLUT1, glucose transporter 1; HO-1, heme oxygenase 1; IGFBP1, insulin-like growth-factor binding protein 1; LDHA, lactate dehydrogenase A; iNOS, inducible nitric oxide synthase; PFKL, phosphofructokinase L; PGK1, phosphoglycerate kinase 1; PKM, pyruvate kinase M; TF, transferrin; TR, transferring receptor; VEGF, vascular endothelial growth factor; FLT-1, VEGF receptor. Note, in the above table, several sequences has “CACG” that is the complementary sequence of “CGTG”.

**Table 3:** The set of 105 common patterns from 15 training genes.

AAAC	AGGC	CCCTT	CTTC	GCGA	GGGAG	TCCA
AACT	AGGGA	CCGGG	CTTG	GCGT	GGGC	TCCCC
AAGCA	ATCC	CCTC	GAAA	GCTA	GGGGC	TCCG
AAGG	CAAG	CCTG	GAAC	GCTC	GGGT	TCCTG
AAGT	CACA	CCTT	GACC	GCTGG	GGTC	TCTT
ACAC	CACC	CGGA	GAGCC	GCTTC	GGTG	TGAC
ACAG	CACG	CGGG	GAGGA	GGAA	GTCCT	TGAG
ACCC	CAGA	CGTG	GAGT	GGAC	GTGA	TGCCT
ACCT	CAGCA	CTAG	GATC	GGAGC	GTGCT	TGCG
ACGC	CAGCC	CTAT	GATG	GGAT	GTGT	TGCTG
AGAA	CAGGC	CTCA	GCAC	GGCC	TAAA	TGGC
AGAGC	CCAGC	CTCCC	GCAG	GGCG	TAGGG	TGGG
AGCAG	CCAT	CTGC	GCCA	GGCTG	TATA	TGTG
AGCCT	CCCAG	CTGGC	GCCC	GGCTT	TCAGG	TTCA
AGGAC	CCCCA	CTGT	GCCT	GGGAA	TCAT	TTCT

**Table 4:** Average case complexity for the proposed algorithms.

Complexity		Alg. 1	Alg. 2	Alg. 3
Time	Construction	*O*(*n_s_l_s_*) *O*(*|S_d_|*)	*O*(*n_s_l_s_* + *n_p_l_p_*)	*O*(*n_p_l_p_*) = *O*(*|S_c_|*)
	Search	*O*(*n_s_n_p_l_p_*) *O*(*n_s_|S_c_|*)	*O*(*n_p_l_p_*)	*O*(*n_s_l_s_*) = *O*(*|S_d_|*)
	Total	*O*(*n_s_l_s_* + *n_s_n_p_l_p_*) ≈ *O*(*n_s_l_s_*)	*O*(*n_s_l_s_* + *n_p_l_p_*) ≈*O*(*n_s_l_s_*)	*O*(*n_s_l_s_* + *n_p_l_p_*) ≈*O*(*n_s_l_s_*)
Space	Construction	*O*(*w_s_*)	*O*(*n_s_l_s_* + *n_p_l_p_*)	*O*(*n_p_l_p_*)
	Search	*O*(*l_p_*)	*O*(*l_p_*)	*O*(*w_s_*)
	Total	*O*(*w_s_* + *l_p_*) ≈ *O*(*w_s_*)	*O*(*n_s_l_s_* + *n_p_l_p_*) ≈ *O*(*n_s_l_s_*)	*O*(*n_p_l_p_*) + *O*(*w_s_*).≈ *O*(*w_s_*)

* Average case complexity for the proposed algorithms (*n_s_* = number of sequences in the database, *n_p_* = number of common patterns, *i_s_* = average length of a sequence, *i_p_* = average length of a pattern, *w_s_* *=* number of characters processed at one time (size of the sliding window), *s_c_* is the concatenation of all the multiple patterns, and *s_d_* is the concatenation of all the sequences in the database).

**Table 5:** Comparative results for Algorithm 1 and Algorithm 2.

DB	Sequences	Size (MB)	Avg./Seq (symbols)	Avg. speed (sequences/min)		Avg. speed (KB/min)	
				Alg. 1	Alg. 2	Alg. 1	Alg. 2
1st/6	346,466	643	1,856	216	521	401	967
2nd/6	346,464	1511	4,360	196	279	851	1,216
3rd/6	346,464	2125	6,134	195	216	1,196	1,325
4th/6	346,464	2691	7,766	169	169	1,312	1,312
5th/6	346,464	1638	4,728	189	299	894	1,414
6th/6	346,464	1661	4,793	197	285	944	1,366
Total	2,078,786	10,268	4,940	192	262	948	1,294

**Table 6:** The 25 HIF-1 known target genes in the final output.

Gene name	Accession^#^	ID
ALDA	X06351*, X12447, J05517	1
α_1B_AR	D32045, AF116943*, X51585	2
DEC1	AB043885	3
cyclin G2	AF549495	4
ET-1	S76970*	5
EPO	M11319	6
HO-1	U70472*	7
c-met	AF046925*	8
IGFBP1	AY434089*, M74587, M59316	9
LDHA	U13679*, Y00309	10
PFKL	M61210*	11
iNOS	AJ308545, L23806 (AY445095*)	12
FLT-1	AJ224863*	13
ENO1	X16287*	14
p21(WAF)	U24170	15
p35srj	AF129290	16
ETS-1	L20682	17
TFR	X04664*	18
VEGF	M63971, AF095785*	19
ADM	S73906*, D78349	20
GLUT1	U82755*	21
PGK	X15339, AF335419	22
TGFα	AL732598	23
Nip3	AF283504	24
trefoil factor	AB038162	25

* Indicates the training set of 15 HIF-1 target genes.

**Table 7:** The 17 putative novel targets identified to be upregulated by HIF-1 or hypoxia based on microarray data through literature search.

Accession^#^	Gene name	Ref.
AY282416	Interleukin 8	50
M11567	Angiogenin	50
AY339617	carbohydrate sulfotransferase 1	51
AL121586	fer-1-like 4 (*C. elegans*)	51
AF050157	hypothetical protein	51
AF157623	serine protease	51
AJ400879	ribosomal protein L27a	51
AY428630	neuroblastoma RAS viral oncogene	51
U06950	tumor necrosis factor, lymphotoxin	51
AK038789	B-cell CLL/lymphoma	51
AK549495	cyclin-dependent kinase	51
AY149618	heat shock 70 kDa protein 1A	53
AY149619	heat shock 70 kDa protein 1A	53
NM_003670	BHLHB2	52
NM_017817	RAS oncogene	52
NM_009320	solute carrier family 6	53
AF055066	MHC class I	53
